# Psychological Health Issues of Medical Staff During the COVID-19 Outbreak

**DOI:** 10.3389/fpsyt.2021.611223

**Published:** 2021-04-30

**Authors:** Jun Xie, Qi Liu, Xiaobing Jiang, Upasana Manandhar, Zhen Zhu, Yuanyuan Li, Bo Zhang

**Affiliations:** ^1^Department of Dermatology, Zhongnan Hospital of Wuhan University, Wuhan University, Wuhan, China; ^2^Department of Maternal and Child Health, Ministry of Education (MOE) Key Lab of Environment and Health, School of Public Health, Tongji Medical College, Huazhong University of Science and Technology, Wuhan, China; ^3^Department of Neurosurgery, Wuhan Union Hospital, Tongji Medical College, Huazhong University of Science and Technology, Wuhan, China; ^4^Department of Orthopedics, Union Hospital, Tongji Medical College, Huazhong University of Science and Technology, Wuhan, China; ^5^Key Laboratory of Environment and Health, Ministry of Education & Ministry of Environmental Protection, State Key Laboratory of Environmental Health, School of Public Health, Tongji Medical College, Huazhong University of Science and Technology, Wuhan, China

**Keywords:** COVID-19, psychological health, medical staff, epidemics, epidemiology

## Abstract

**Background:** The outbreak of novel coronavirus disease 2019 (COVID-19) has caused public panic and psychological health problems, especially in medical staff. We aimed to investigate the psychological effect of the COVID-19 outbreak on medical staff.

**Methods:** A cross-sectional study was conducted to examine the psychological impact of medical staff working in COVID-19 designated hospitals from February to March 2020 in China. We assessed psychological health problems using the Symptom Check List 90 (SCL-90).

**Results:** Among 656 medical staff, 244 were frontline medical staff and 412 general medical staff. The prevalence of psychological health problems was 19.7%. The SCL-90 scores in frontline medical staff were significantly higher than that in general medical staff (mean: 141.22 vs. 129.54, *P* < 0.05). Furthermore, gender [odds ratio (OR) = 1.53, 95% CI = (1.02, 2.30), *P* = 0.042 for female vs. male] and the burden of current work [OR = 7.55, 95% CI = (3.75, 15.21), *P* < 0.001 for high burden; OR = 2.76, 95% CI = (1.80, 4.24), *P* < 0.001 for moderate burden vs. low burden] were associated with increased risk of poor psychological status.

**Conclusions:** Medical staff experienced a high risk of psychological health problems during the outbreak of COVID-19, especially for frontline medical staff. Psychological health services are expected to arrange for medical staff in future unexpected infectious disease outbreaks.

## Introduction

In 2019, the novel coronavirus disease 2019 (COVID-19) epidemic occurred in Wuhan, Hubei, China, and subsequently attracted worldwide attention (1). Within 2 months, the outbreak had spread to more than one hundred and thirty countries in the world, and the number of confirmed cases grew quickly to 63,000. On January 30, 2020 (Beijing Time), the World Health Organization (WHO) has declared the current COVID-19 outbreak as “a global public health emergency of international concern.” Due to the rapid spread and high mortality of the disease, the COVID-19 epidemic caused considerable panic and anxiety worldwide. Therefore, it is an urgent need to explore the possible psychosocial impacts of the COVID-19 outbreak.

Medical staff is at particularly high risk of psychological health problems when facing these unprecedented challenges. The disease appears to transmit via close person-to-person contact (2). Due to a lack of adequate personal protective equipment in patient care areas, medical staff experienced a great risk of COVID-19 infection (3). Their job puts them at increased risk of exposure to the COVID-19 epidemic. The Information Office of the State Council held a press conference in Wuhan on March 6, 2020, reporting that more than 3,000 medical staff were infected with coronavirus pneumonia in Hubei Province, of which 40% were hospital infection and 60% were community infection. Moreover, medical staff was carrying a large burden in the clinical treatment and public prevention efforts. The heavy burden of medical work may also be a risk factor for poor psychological health. Additionally, there are many identifiable reasons for the unbearable psychological distress, such as fear of bringing the virus to their home (4). Therefore, the challenges and stress they had experienced could trigger a series of mental disorders, including anxiety and depression, which may lead to more harm that exceeds the consequences of the 2019-nCoV epidemic itself.

The global pandemic of COVID-19 poses an unprecedented threat to the world. Because of the high infectivity and unclear nature of the virus, everyone was in a state of great panic, especially for the high-risk medical staff (5). To improve understanding of the psychological impact of exposure to a fast-spreading, life-threatening infectious disease among medical staff, and provide better guidance for the world to cope with the outbreak, we conducted the present study. We sought to examine the psychological impact among medical staff facing the COVID-19 epidemic in China, the most heavily affected country in the world, and find out related risk and protective factors.

## Methods

### Participants

In order to evaluate current psychological health among clinical staff during the COVID-19 epidemic, we conducted an internet-based cross-sectional study from February 17th to March 8th, 2020, China. Because the virus hinders face-to-face communication, online anonymous questionnaire is the safest choice for data collection. We adopted the online mode to questionnaire in Wuhan and other areas of the country. Participants were invited to participate in the online survey if they were medical staff from the designated hospitals for novel coronavirus treatment in China, especially those who were involved in the events as frontline healthcare workers or working as general medical staff since December 2019. After giving detailed informed consent, 656 medical staff approved and completed the online questionnaires. The study was conducted following the Declaration of Helsinki, and the Ethics Committee of Union Hospital, Tongji Medical College, Huazhong University of Science and Technology approved the protocol [No. (2020) 0029].

We collected information about the psychological health status among the participants during the outbreak and remission of the COVID-19 epidemic, including demographic characteristics of living in Wuhan (yes and no), age (years), gender (male and female), work-related stress: work (frontline and general medical staff), the frequency of work (per week) (1–2 days, 3–5 days, and more than 5 days), the burden of current medical work (low, moderate, and high), rest place (at home, at the hospital, and at the hotel), and family-related stress: spouse's work (medical staff, community workers or other works that could contact with novel coronavirus pneumonia patients, and having a rest at home), the number of minor children (0, 1, ≥2), the caregivers of children (parents, grandparents, other relatives and friends, and no one), having caregivers to take care of their parents (yes and no), and relatives, friends or neighbors got COVID-19 (yes and no). Frontline medical staff was defined as having contact with patients who were confirmed or suspected cases of COVID-19. General medical staff was defined as having no contact with fever patients in the work.

### Psychological Health Assessment

As a reliable and valid tool for psychological evaluation, the Symptom Check List 90 (SCL-90) is widely applied in many fields (6), including mental health assessment in medical and health professionals (7–9). The SCL-90 was introduced in China and revised according to the social and cultural background of China in 1984 (10). The norm of SCL-90 was established by Jin et al. among 1,388 healthy adults in 13 regions of China (11). The SCL-90 scale consists of 90 self-reported items, which are divided into nine subscales (somatization, obsessive-compulsive, interpersonal sensitivity, depression, anxiety, anger hostility, phobic anxiety, paranoid ideation, and psychoticism), as well as an additional scale to measure disturbances in appetite and sleep. Each item is scored using a 5-point scale (1~5; 1 = Not at all; 5 = Extremely) to measure the symptoms experienced in the past 7 days. We add up all items to calculate the total score of psychological distress. The higher the SCL-90 scores, the more severe psychopathologic symptoms. The total score of the SCL-90 scale is above 160, which indicates that the subject has positive psychological health problems (12).

### Data Analysis

First, we examined the frequency distributions of sociodemographic characteristics, work-related factors, and family-related factors in the study. Categorical variables were shown as number (%) and continuous variables were expressed as the mean and standard deviation (SD). Second, we applied the chi-square test, *t-*test and one-way analysis of variance to test the distribution differences of SCL-90 scores and psychological health problem between frontline and general medical staff. Furthermore, all variables were included in the stepwise linear regression models (entry/removal criteria of *P* = 0.05/0.1) and forward stepwise logistic regression models for further analysis to identify potential risk factors that were related to self-reported psychopathology. In this study, all statistical analyses were performed using the Statistical Package for the Social Sciences (SPSS), version 22.0 (SPSS Inc., Chicago, IL, USA), with all *P*-values <0.05 indicating statistically significant.

## Results

### Demographic and Social Life-Related Characteristics of the Participants

Table 1 shows the descriptive characteristics of the participants. There were 656 medical staff included in the study, of which 244 were frontline medical staff and 412 general medical staff. Among 656 participants, about 321 (48.9%) were in Wuhan during the COVID-19 outbreak and the mean age was 37.28 (SD = 6.36) years old (range 22–69 years old). Approximately half of the respondents were female (*n* = 343; 52.3%) and were working 3–5 days per week (*n* = 312; 47.6%). There were 38 (5.8%) subjects having a high burden of their current workload. In addition, compared with general medical staff, frontline medical staff were more likely to be in Wuhan (75.0 vs. 33.5%), were more likely to work more than 5 days per week (41.0 vs. 36.7%), had a lower burden of their current medical work (7.4 vs. 4.9%), were less took a rest at home (32.4 vs. 92.7%), were fewer medical staff for their spouse (33.6 vs. 39.6%), have a higher proportion of confirmed or suspected patients in their relatives, friends or neighbors (34.0 vs. 21.4%). A total of 129 (19.7%) medical staff were psychologically distressed. The prevalence of psychological health problems was 23.8% in frontline medical staff, which was significantly higher than in general medical staff (17.2%, *P* = 0.042).

**Table 1:** The descriptive characteristics of the participants.

**Variables**	**All medical staff**	**Frontline medical staff (*n* = 244)**	**General medical!!break staff (*n* = 412)**	***P***
**Demographic characteristics**				
Living in Wuhan				<0.001
No	335 (51.1)	61 (25.0)	274 (66.5)	
Yes	321 (48.9)	183 (75.0)	138 (33.5)	
Age (years)	37.28 ± 6.36	36.27 ± 5.92	37.88 ± 6.56	0.002
Gender				0.299
Male	313 (47.7)	110 (45.1)	230 (49.3)	
Female	343 (52.3)	134 (54.9)	209 (50.7)	
**Work-related stress**				
The frequency of work (per week)				<0.001
1–2 days	93 (14.2)	16 (6.6)	77 (18.7)	
3–5 days	312 (47.6)	128 (52.5)	184 (44.7)	
More than 5 days	251 (38.2)	100 (41.0)	151 (36.7)	
The burden of current work				<0.001
Low	435 (66.3)	134 (54.9)	301 (73.1)	
Moderate	183 (27.9)	92 (37.7)	91 (22.1)	
High	38 (5.8)	18 (7.4)	20 (4.9)	
Rest place				<0.001
At home	461 (70.3)	79 (32.4)	382 (92.7)	
At the hospital	48 (7.3)	35 (14.3)	13 (3.2)	
At the hotel	147 (22.4)	130 (53.3)	17 (4.1)	
**Family-related stress**				
Spouse's work				0.016
Medical staff	245 (37.4)	82 (33.6)	163 (39.6)	
Community workers or other works that could contact with COVID-19 patients	94 (14.3)	27 (11.1)	67 (16.3)	
Having a rest at home	317 (48.3)	135 (55.3)	182 (44.1)	
The number of minor children				0.377
0	130 (19.8)	46 (18.9)	84 (20.4)	
1	332 (50.6)	132 (54.1)	200 (48.5)	
≥2	194 (29.6)	66 (27.0)	128 (31.1)	
The caregivers of children				0.563
Parents	225 (34.3)	91 (37.3)	134 (32.5)	
Grandparents	278 (42.4)	99 (40.6)	179 (43.4)	
Other relatives and friends	32 (4.9)	13 (5.3)	19 (4.6)	
No one	121 (18.4)	41 (16.8)	80 (19.4)	
Having caregivers to take care of their parents				0.911
No	423 (64.5)	158 (64.8)	265 (64.3)	
Yes	233 (35.5)	86 (35.2)	147 (35.7)	
Relatives, friends or neighbors got COVID-19				<0.001
No	485 (73.9)	161 (66.0)	324 (78.6)	
Yes	171 (26.1)	83 (34.0)	88 (21.4)	
Psychological health problems				0.042
No	527 (80.3)	186 (76.2)	341 (82.8)	
Yes	129 (19.7)	58 (23.8)	71 (17.2)	

*COVID-19, novel coronavirus pneumonia 2019*.

### Psychological Health Status

The distribution of SCL-90 scales score for the participants are described in Table 2. The total SCL-90 scores in medical staff (mean = 133.88) were significantly higher than the normal scores of SCL-90 in China (mean = 129.96) (*P* < 0.05). Concerning the subscale scores, there was a statistically significant increment in the scores for obsessive-compulsive, interpersonal sensitivity, depression, anxiety, phobic anxiety, and psychoticism among medical staff compared with the national norm (*P* < 0.05 for all of these subscales). Furthermore, we observed significant differences in SCL-90 scores between frontline medical staff (mean = 141.22) and general medical staff (mean = 129.54). In comparison with the SCL-90 scores among general medical staff, frontline medical staff reported a significant increment SCL-90 subscale scores for somatization, obsessive-compulsive, depression, anxiety, and psychoticism (all *P* < 0.05).

**Table 2:** The distribution of SCL-90 scores in the study.

**SCL-90 scales**	**All medical staff**	**Frontline medical staff**	**General medical staff**	**The norm scores in China**
Total score	133.88 ± 50.95^a^	141.22 ± 55.27^a^^,^^b^	129.54 ± 47.74^b^	129.96 ± 38.76
**Subscale score**				
Somatization	1.41 ± 0.55	1.53 ± 0.63^a^^,^^b^	1.34 ± 0.48^b^	1.37 ± 0.48
Obsessive-compulsive	1.70 ± 0.68^a^	1.80 ± 0.71^a^^,^^b^	1.64 ± 0.66^b^	1.62 ± 0.58
Interpersonal sensitivity	1.49 ± 0.65^a^	1.54 ± 0.70^a^	1.46 ± 0.62^a^	1.65 ± 0.51
Depression	1.58 ± 0.66^a^	1.66 ± 0.68^a^^,^^b^	1.53 ± 0.64^b^	1.50 ± 0.59
Anxiety	1.49 ± 0.63^a^	1.59 ± 0.70^a^^,^^b^	1.43 ± 0.58^b^	1.39 ± 0.43
Anger hostility	1.48 ± 0.63	1.52 ± 0.67	1.47 ± 0.60	1.48 ± 0.56
Phobic anxiety	1.35 ± 0.57^a^	1.40 ± 0.61^a^	1.33 ± 0.55^a^	1.23 ± 0.41
Paranoid ideation	1.40 ± 0.61	1.45 ± 0.66	1.37 ± 0.58^a^	1.43 ± 0.57
Psychoticism	1.33 ± 0.54^a^	1.39 ± 0.58^a^^,^^b^	1.30 ± 0.51^b^	1.29 ± 0.42

*SCL-90, the Symptom Check List 90*.

*Data are shown as mean SCL-90 score ± standard deviation*.

a *P < 0.05 The SCL-90 scores among medical staff vs. the norm scores in China*.

b *P < 0.05 The SCL-90 scores among frontline medical staff vs. the SCL-90 scores among general medical staff*.

### Risk Factors for Psychological Health Problems

First, demographic risk factors associated with SCL-90 scores and psychological health status are depicted in Supplementary Tables 1, 2. As shown in Supplementary Table 1, the score of SCL-90 in the frontline medical staff with higher burden (mean = 221.28) was significantly higher than that in the medical staff with moderate (mean = 152.87) or light burden (mean = 122.46). Similarly, in the general medical staff, the total of SCL-90 in the medical staff with high burden (mean = 186.00) was significantly higher than that in the medical staff with moderate (mean = 143.37) or light burden (mean = 121.60). Furthermore, the SCL-90 score of the general medical staff resting in hospital (mean = 174.69) was significantly higher than that of the medical staff resting at the hotel (mean = 151.29) or at home (mean = 127.03). Compared with these general medical staff having caregivers to take care of their parents, the general medical staff whose parents having nobody taking care of having a higher SCL-90 score (mean = 119.53 vs. 135.09, *P* < 0.05). This is consistent with the distribution of poor psychological status in different demographic and social life related factors (Supplementary Table 2). Then, in order to examine risk factors associated with current psychological health among 656 medical staff during the COVID-19 outbreak, all variables were included for stepwise linear regression analysis (Table 3). Medical staff resident in Wuhan [β = 10.09, 95% CI = (2.91, 17.27), *P* = 0.006], with a high burden of current work [β = 32.04, 95% confidence interval (95% CI) = (25.94, 38.13), *P* < 0.001] and having no body taking care of their parents [β = 9.22, 95% CI = (1.61, 16.82), *P* = 0.018] showed a significantly high SCL-90 score. When restricted to frontline medical staff, we found a significant association between the SCL-90 scores and the burden of current work during the COVID-19 outbreak [β = 41.02, 95% CI = (31.23, 50.80), *P* < 0.001]. Similarly, when restricted to general medical staff, the SCL-90 scores were associated with the burden of current work during the COVID-19 outbreak [β = 24.21, 95% CI = (16.25, 32.16), *P* < 0.001] and having caregivers to take care of their parents [β = 10.65, 95% CI = (1.45, 19.84), *P* = 0.024].

**Table 3:** Risk factors associated with mental health status in linear regression models.

	**β**	**95% CI**	***P***
**All medical staff**			
Living in Wuhan	10.09	(2.91, 17.27)	0.006
The burden of current work	32.04	(25.94, 38.13)	<0.001
Having caregivers to take care of their parents	9.22	(1.61, 16.82)	0.018
**Frontline medical staff**			
The burden of current work	41.02	(31.23, 50.80)	<0.001
**General medical staff**			
The burden of current work	24.21	(16.25, 32.16)	<0.001
Having caregivers to take care of their parents	10.65	(1.45, 19.84)	0.024

*CI, confidence interval; COVID-19, novel coronavirus pneumonia 2019*.

In logistic regression analysis, gender [odds ratio (OR) = 1.53, 95% CI = (1.02, 2.30), *P* = 0.042 for female vs. male] and the burden of current work [OR = 7.55, 95% CI = (3.75, 15.21), *P* < 0.001 for high burden; OR = 2.76, 95% CI = (1.80, 4.24), *P* < 0.001 for moderate burden vs. low burden] at inclusion were significantly increased the risk of poor psychological health. In stratified analysis, the associations between poor psychological problems and the burden of current work during the COVID-19 outbreak were significant in frontline medical staff [OR = 20.38, 95% CI = (6.28, 66.19), *P* < 0.001 for high burden; OR = 3.53, 95% CI = (1.77, 7.04), *P* < 0.001 for moderate burden vs. low burden] and general medical staff [OR = 3.53, 95% CI = (1.32, 9.46), *P* < 0.001 for high burden; OR = 2.38, 95% CI = (1.33, 4.29), *P* < 0.001 for moderate burden vs. low burden] (Table 4).

**Table 4:** Risk factors associated with mental health problems in logistic regression models.

	**OR**	**95% CI**	***P***
**All medical staff**			
Gender			
Male	Ref		
Female	1.53	(1.02, 2.30)	0.042
The burden of current work			
Low	Ref		
Moderate	2.76	(1.80, 4.24)	<0.001
High	7.55	(3.75, 15.21)	<0.001
**Frontline medical staff**			
The burden of current work			
Low	Ref		
Moderate	3.53	(1.77, 7.04)	<0.001
High	20.38	(6.28, 66.19)	<0.001
**General medical staff**			
The burden of current work			
Low	Ref		
Moderate	2.38	(1.33, 4.29)	0.004
High	3.53	(1.32, 9.46)	0.012
Relatives, friends or neighbors got COVID-19			
No		Ref	
Yes	1.90	(1.05, 3.43)	0.034

*COVID-19, novel coronavirus pneumonia 2019; OR, odds ratio*.

## Discussion

Since the end of 2019, the Novel coronavirus pneumonia 2019 (COVID-19) outbroke in Wuhan, China, thereby brought an unprecedented challenge to China's medical health care system. In the present study, the medical staff working in novel coronavirus pneumonia designated hospitals were invited to participate in the cross-sectional internet-based survey. A total of 656 medical staff completed the questionnaire from February to March 2020, including 244 frontline medical staff and 412 general medical staff.

We found that medical staff was psychologically distressed during the outbreak of the COVID-19 epidemic, especially for frontline medical staff. In regarding to the nine subscales, the score of obsessive-compulsive, interpersonal sensitivity, depression, anxiety, phobic anxiety, and psychoticism was significantly increased than that of general populations. These findings indicated that in the face of epidemic, the psychological health of medical staff was affected in all aspects, which was consistent with the findings during the 2003 severe acute respiratory syndrome (SARS) outbreak (13–17). According to previous studies, health care workers who have more access to SARS patients were at high risk of depression and anxiety (18–23). In addition, Cai et al. conducted a study in Jiangsu Province among 1,521 health care workers to investigate the psychological abnormality in health care workers battling the COVID-19 epidemic, and reported that the prevalence of psychological abnormality was 14.1% (24).During February 19 to March 6, 2020, an online survey involving 2,182 Chinese subjects shown that medical health workers had a higher total scores of SCL-90-R obsessive-compulsive symptoms compared with non-medical health workers (8). What's more, regarding psychological distress among health care workers, there were several reviews systematically reviewed the current evidence (25–30). It revealed that higher levels of depression/depressive symptoms, anxiety, poor sleep quality and obsessive–compulsive disorder symptoms were reported in medical health workers compared to non-medical staff. We found a great variability in the prevalence estimates, probably due to different cut-off scores used to identify cases or to the use of heterogeneous instruments (e.g., GAD-7, SDS, SAS, SCL-90-R). Nevertheless, as a highly exposed group, a higher prevalence of psychological symptoms among health care workers during COVID-19 are still worthy of public attention.

In the present study, the high burden of current work is the main risk factor of poor psychological status among medical staff during the COVID-19 outbreak. When novel coronavirus pneumonia occurred in Wuhan in the end of 2019, the epidemic spread rapidly nationwide in a short time, which resulted in obvious shortage of medical staff. As a result, many medical staff have to work continuously for long hours every day, and frontline health workers were bearing significantly increased workload. That might lead to high levels of psychological distress (31).

Worrying about the take care of their parents was also an important risk factor for psychological health issues of medical staff. The average age of the medical staff who participated in the survey was 37.28 years old. It was speculated that most of their parents were in the old age stage (over 65 years old). These medical staff who need to take care of their parents may have great psychological pressure.

Gender may be a significant factor for psychological stress during the epidemic, and female medical staff was at great risk of poor psychological health. Several studies among medical staff during the SARS-CoV-2 pandemic have indicated that women reported more severe symptoms of depression, anxiety, and distress than men (32–34). In general, women's physical ability is not as good as men's, and the excessive workload inevitably leads to women's greater anxiety than men. Furthermore, these mental disorders are generally more frequent among women (35). Considering that, particular attention is warranted regarding the mental health well-being of female front-line medical workers.

In the present study, we conducted investigations in the stage of the COVID-19 epidemic to evaluate the psychological health status of medical staff. This study has highlighted the psychological impact of COVID-19 on medical staff, especially for frontline medical staff. In face of public health emergencies, we should pay close attention to improve the psychological coping ability of medical staff under pressure and provide better psychological guidance through mental health lectures and psychological counseling. An informal psychological support group can help to stabilize our mind and maintain a positive and optimistic state to copy with all kinds of emergencies, so as to reduce the risk of anxiety, depression and other mental disorders (36). In addition, taking actively care for the family members of medical staff and let them work at ease appeared to be important. It is recommended that managers should strengthen communication, understand the difficulties they encounter in work and life, and provide timely help to reduce their pressure of life.

There are some limitations existing in the study. First, we choose the normal score of SCL-90 in China as the controls. The Chinese norm was established more than 20 years ago, which may be unsuitable for the current study (12). Additionally, some possible factors associated with psychological disorders, such as isolation, stigma, illness, were not identified in the analysis, although we have included related risk factors in the aspects of demographic characteristics, work-related stress and family-related stress. Furthermore, this present study adopted the self-administered questionnaires. Reliance on self-report data may affect the accuracy of the results. Finally, a cross-sectional study was used to evaluate the psychological status among medical staff during the COVID-19 epidemic. Considering the lack of investigation results before the epidemic, it is hard to evaluate the psychological status of medical staff before the COVID-19 epidemic. In view of the continuing impact that the pandemic event entails on health care workers, we will conduct in-depth follow-up studies to explore the long-term psychological impact of medical staff when the global emergency is over.

## Conclusions

In conclusion, the study was conducted to explore the psychological health effect of the COVID-19 outbreak on medical staff, and it was found that frontline medical staff was more psychologically distressed during the COVID-19 outbreak. Our results support more psychological intervention measures in serious emergency health events to reduce the damage of medical staff.

## Data Availability Statement

The raw data supporting the conclusions of this article will be made available by the authors, without undue reservation.

## Ethics Statement

The studies involving human participants were reviewed and approved by the Ethics Committee of Union Hospital, Tongji Medical College, Huazhong University of Science and Technology [No. (2020) 0029]. The patients/participants provided their written informed consent to participate in this study.

## Author Contributions

BZ and YL contributed to the conception of the study. JX, XJ, UM, and ZZ collected the data. JX and QL performed the data analyses and wrote the manuscript. BZ and YL revised the draft of the paper. All authors have read and agreed to the published version of the manuscript.

## Conflict of Interest

The authors declare that the research was conducted in the absence of any commercial or financial relationships that could be construed as a potential conflict of interest. The reviewer NX declared a shared affiliation, though no other collaboration, with the authors, QL, XJ, ZZ, YL, and BZ, to the handling Editor.
