# Supplementary material for: Psychological Health Issues of Medical Staff During the COVID-19 Outbreak
Source: Front Psychiatry. 2021 Apr 30;12:611223. doi: 10.3389/fpsyt.2021.611223 (PMC8119643; doi:10.3389/fpsyt.2021.611223)
Supplement: Supplementary file 1 [file Table_1.DOCX]

Supplementary Material

# Supplementary Tables

**Supplement table S1**. Descriptive statistics of the SCL-90 scores in the study.

| **Variables** | **Frontline medical staff** | |  | **General medical staff** | |
| --- | --- | --- | --- | --- | --- |
|  | **SCL-90** | ***P*** |  | **SCL-90** | ***P*** |
| Living in Wuhan |  | 0.147 |  |  | 0.249 |
| No | 132.33±46.06 |  |  | 127.61±48.39 |  |
| Yes | 144.18±57.83 |  |  | 133.36±46.37 |  |
| Gender |  | 0.281 |  |  | 0.390 |
| Male | 137.00±52.89 |  |  | 127.48±42.79 |  |
| Female | 144.68±57.12 |  |  | 131.53±52.13 |  |
| The frequency of work (per week) | | 0.163 |  |  | 0.051 |
| 1-2 days | 127.31±31.75 |  |  | 127.17±43.81 |  |
| 3-5 days | 137.03±46.85 |  |  | 124.43±39.30 |  |
| More than 5 days | 148.80±66.47 |  |  | 136.96±57.43 |  |
| The burden of current work | | <.0001 |  |  | <.0001 |
| Low | 122.46±30.95 |  |  | 121.60±36.01 |  |
| Moderate | 152.87±54.09 |  |  | 143.37±55.28 |  |
| High | 221.28±101.09 |  |  | 186.00±95.29 |  |
| Rest place |  | 0.768 |  |  | < 0.001 |
| At home | 138.41±47.09 |  |  | 127.03±42.38 |  |
| At the hospital | 146.54±46.11 |  |  | 174.69±46.14 |  |
| At the hotel | 141.49±61.97 |  |  | 151.29±70.80 |  |
| Spouse's work |  | 0.526 |  |  | 0.356 |
| Medical staff | 143.96±51.83 |  |  | 133.71±48.61 |  |
| Community workers or other works that could contact with novel coronavirus pneumonia patients | 149.48±67.98 |  |  | 127.22±53.33 |  |
| Having a rest at home | 137.90±54.68 |  |  | 126.65±44.70 |  |
| The number of minor children | | 0.586 |  |  | 0.218 |
| 0 | 148.83±72.21 |  |  | 132.27±59.83 |  |
| 1 | 139.57±50.18 |  |  | 132.30±44.50 |  |
| ≥2 | 139.21±51.82 |  |  | 123.42±43.35 |  |
| The caregivers of children | | 0.149 |  |  | 0.312 |
| Parents | 135.33±56.27 |  |  | 123.54±36.18 |  |
| Grandparents | 139.05±42.86 |  |  | 133.63±52.78 |  |
| Other relatives and friends | 166.54±50.51 |  |  | 126.79±37.37 |  |
| No one | 151.49±75.91 |  |  | 131.09±54.37 |  |
| Having caregivers to take care of their parents | | 0.064 |  |  | 0.001 |
| No | 146.04±58.44 |  |  | 135.09±53.11 |  |
| Yes | 132.35±48.00 |  |  | 119.53±34.04 |  |
| Relatives, friends or neighbors got COVID-19 | | 0.744 |  |  | 0.175 |
| No | 140.39±54.71 |  |  | 127.87±47.40 |  |
| Yes | 142.83±56.65 |  |  | 135.66±48.76 |  |

SCL-90: the Symptom Check List 90; COVID-19: novel coronavirus pneumonia 2019.

**Supplement table S2**. Descriptive statistics of mental health problems prevalence in the study.

| **Variables** | **Frontline medical staff** | | |  | **General medical staff** | | |
| --- | --- | --- | --- | --- | --- | --- | --- |
|  | **SCL-90-positive** | **SCL-90-negative** | ***P*** |  | **SCL-90-positive** | **SCL-90-negative** | ***P*** |
| All populations | 58 (100.0) | 186 (100.0) | *-* |  | 71 (100.0) | 341 (100.0) | *-* |
| Living in Wuhan |  |  | 0.385 |  |  |  | 0.244 |
| No | 12 (20.7) | 49 (26.3) |  |  | 43 (60.6) | 231 (67.7) |  |
| Yes | 46 (79.3) | 137 (73.7) |  |  | 28 (39.4) | 110 (32.3) |  |
| Gender |  |  | 0.120 |  |  |  | 0.299 |
| Male | 21 (36.2) | 89 (47.8) |  |  | 31 (43.7) | 172 (50.4) |  |
| Female | 37 (63.8) | 97 (52.2) |  |  | 40 (56.3) | 169 (49.6) |  |
| The frequency of work (per week) |  |  | 0.410 |  |  |  | 0.638 |
| 1-2 days | 2 (3.4) | 14 (7.5) |  |  | 11 (15.5) | 66 (19.4) |  |
| 3-5 days | 29 (50.0) | 99 (53.2) |  |  | 31 (43.7) | 153 (44.9) |  |
| More than 5 days | 27 (46.6) | 73 (39.2) |  |  | 29 (40.8) | 122 (35.8) |  |
| The burden of current work |  |  | <.0001 |  |  |  | 0.001 |
| Low | 16 (27.6) | 118 (63.4) |  |  | 40 (56.3) | 261 (76.5) |  |
| Moderate | 29 (50.0) | 63 (33.9) |  |  | 24 (33.8) | 67 (19.6) |  |
| High | 13 (22.4) | 5 (2.7) |  |  | 7 (9.9) | 13 (3.8) |  |
| Rest place |  |  | 0.499 |  |  |  | 0.043 |
| At home | 17 (29.3) | 62 (33.3) |  |  | 61 (85.9) | 321 (94.1) |  |
| At the hospital | 11 (19.0) | 24 (12.9) |  |  | 5 (7.0) | 8 (2.3) |  |
| At the hotel | 30 (51.7) | 100 (53.8) |  |  | 5 (7.0) | 12 (3.5) |  |
| Spouse's work |  |  | 0.618 |  |  |  | 0.219 |
| Medical staff | 17 (29.3) | 65 (34.9) |  |  | 34 (47.9) | 129 (37.8) |  |
| Community workers or other works that could contact with novel coronavirus pneumonia patients | 8 (13.8) | 19 (10.2) |  |  | 8 (11.3) | 59 (17.3) |  |
| Having a rest at home | 33 (56.9) | 102 (54.8) |  |  | 29 (40.8) | 153 (44.9) |  |
| The number of minor children |  |  | 0.993 |  |  |  | 0.073 |
| 0 | 11 (19.0) | 35 (18.8) |  |  | 16 (22.5) | 68 (19.9) |  |
| 1 | 31 (53.4) | 101 (54.3) |  |  | 41 (57.7) | 159 (46.6) |  |
| ≥2 | 16 (27.6) | 50 (26.9) |  |  | 14 (19.7) | 114 (33.4) |  |
| The caregivers of children |  |  | 0.065 |  |  |  | 0.534 |
| Parents | 21 (36.2) | 70 (37.6) |  |  | 18 (25.4) | 116 (34.0) |  |
| Grandparents | 20 (34.5) | 79 (42.5) |  |  | 35 (49.3) | 144 (42.2) |  |
| Other relatives and friends | 7 (12.1) | 6 (3.2) |  |  | 3 (4.2) | 16 (4.7) |  |
| No one | 10 (17.2) | 31 (16.7) |  |  | 15 (21.1) | 65 (19.1) |  |
| Having caregivers to take care of their parents |  |  | 0.650 |  |  |  | 0.364 |
| Yes | 19 (32.8) | 67 (36.0) |  |  | 22 (31.0) | 125 (36.7) |  |
| No | 39 (67.2) | 119 (64.0) |  |  | 49 (69.0) | 216 (63.3) |  |
| Relatives, friends or neighbors got COVID-19 |  |  | 0.069 |  |  |  | 0.063 |
| Yes | 14 (24.1) | 69 (37.1) |  |  | 21 (29.6) | 67 (19.6) |  |
| No | 44 (75.9) | 117 (62.9) |  |  | 50 (70.4) | 274 (66.5) |  |

SCL-90: the Symptom Check List 90; COVID-19: novel coronavirus pneumonia 2019.

SCL-90-positive indicates that the total score of the SCL-90 scale is above 160. *P* value was calculated by chi-square analysis to explore the distribution differences of psychological problems in different factors.
